# Allopurinol use and the risk of acute cardiovascular events in patients with gout and diabetes

**DOI:** 10.1186/s12872-017-0513-6

**Published:** 2017-03-14

**Authors:** Jasvinder A. Singh, Rekha Ramachandaran, Shaohua Yu, Jeffrey R. Curtis

**Affiliations:** 1 Medicine Service, VA Medical Center, 700 19th St S, Birmingham, AL 35233 USA; 2 Department of Medicine at School of Medicine, University of Alabama at Birmingham, 510, 20th street South, Faculty office tower (FOT), Birmingham, AL 35294 USA; 3 Division of Epidemiology at School of Public Health, University of Alabama at Birmingham, 1720 Second Ave. South, Birmingham, AL 335294-0022 USA; 4 Department of Orthopedic SurgeryMayo Clinic College of Medicine, 200 1st St SW, Rochester, MN 55905 USA

**Keywords:** Allopurinol, Acute cardiovascular events, Gout, Diabetes, Myocardial infarction, Stroke, Predictors

## Abstract

**Background:**

Few studies, if any, have examined cardiovascular outcomes in patients with diabetes and gout. Both diabetes and gout are risk factors for cardiovascular disease. The objective of this study was to examine the effect of allopurinol on the risk of incident acute cardiovascular events in patients with gout and diabetes.

**Methods:**

We used the 2007–2010 Multi-Payer Claims Database (MPCD) that linked health plan data from national commercial and governmental insurances, representing beneficiaries with United Healthcare, Medicare, or Medicaid coverage. In patients with gout and diabetes, we assessed the current allopurinol use, defined as a new filled prescription for allopurinol, as the main predictor of interest. Our outcome of interest was the occurrence of the first Incident hospitalized myocardial infarction (MI) or stroke (composite acute cardiovascular event), after which observations were censored. We employed multivariable-adjusted Cox proportional hazards models that simultaneously adjusted for patient demographics, cardiovascular risk factors and other medical comorbidities. We calculated hazard ratios [HR] (95% confidence intervals [CI]) for incident composite (MI or stroke) acute cardiovascular events. We performed sensitivity analyses that additionally adjusted for the presence of immune diseases and colchicine use, as potential confounders.

**Results:**

There were 2,053,185 person days (5621.3 person years) of current allopurinol use and 1,671,583 person days (4576.5 person years) of prior allopurinol use. There were 158 incident MIs or strokes in current and 151 in prior allopurinol users, respectively. Compared to previous allopurinol users, current allopurinol users had significantly lower adjusted hazard of incident acute cardiovascular events (incident stroke or MI), with an HR of 0.67 (95% CI, 0.53, 0.84). Sensitivity analyses, additionally adjusted for immune diseases or colchicine use, confirmed this association.

**Conclusions:**

Current allopurinol use protected against the occurrence of acute cardiovascular events in patients with gout and diabetes. The underlying mechanisms for this potential cardio-protective effect of allopurinol need further exploration.

**Electronic supplementary material:**

The online version of this article (doi:10.1186/s12872-017-0513-6) contains supplementary material, which is available to authorized users.

## Background

Chronic inflammatory conditions, such as rheumatoid arthritis and gout, are associated with significant increase in cardiovascular morbidity and mortality. Several studies have reported a higher risk of cardiovascular disease in patients with gout [1–3]. The evidence supporting the association of hyperuricemia with increased cardiovascular risk [1, 4–7] indicates that this may be at least one potential mechanism for this increased cardiovascular risk in patients with gout.

Allopurinol, a purine analog, acts as a competitive substrate for xanthine oxidase, an enzyme that converts hypoxanthine to xanthine and xanthine into uric acid. Thus, it reduces the level of serum urate and acts as a urate-lowering therapy (ULT). Xanthine oxidase generates superoxide anions and other oxidative free radicals that are associated with oxidative stress [8]. Allopurinol reduces uric acid production and oxidative stress, processes that have been hypothesized to be associated with endothelial dysfunction, inflammation and progression of atherosclerosis [9]. Thus, allopurinol might have beneficial effects on cardiac physiology [10, 11].

Recent studies have debated whether allopurinol has a true cardio-protective effect, i.e., does it reduce the risk of outcomes such as myocardial infarction (MI)? A case-control study with a prevalent user design using the French pharmacoepidemiology database found that allopurinol use did not significantly reduce the risk of non-fatal MI, odds ratio (OR) was 0.80 (95% CI, 0.59, 1.09) [12]. In contrast, in a nested case-control study using Spanish primary care database, current allopurinol use and not past use, was associated with reduction in non-fatal MI with odds ratios of 0.52 (95% CI, 0.33 to 0.83) and 0.99 (95% CI, 0.61, 1.61), respectively [13]. Yet another study of gout patients using Scottish database reported that compared to non-use, allopurinol use was associated with higher (not lower) adjusted hazards of cardiovascular hospitalization (including coronary artery disease (CAD), hypertensive heart disease, heart failure, stroke, other cardiovascular diseases) of 1.25 (95% CI, 1.10, 1.41) [14]. These three studies differed in the main outcome assessed (MI vs. non-fatal MI vs. cardiovascular hospitalization including heart failure) and the country setting (France vs. Spain vs. Scotland), which could have explain the differences in  the results between these three studies.

Key limitations of the previous studies included the use of a prevalent- user design [12, 14], the potential lack of adequate statistical power [12, 13], a short follow-up [13], potential survival bias since non-fatal MI cases were included [12–14], and the non-exclusion of patients with previous MI [14]. A prevalent-user design (used in 2 of the 3 studies) is likely more biased than a new-user design, due to the potential inclusion of factors on the causal pathway and the lack of an ability to capture early events (especially harmful effects) after starting a medication. Incident user design, in which medication exposure time begins with a new start of a medication, allows for more valid treatment comparisons compared to mixing ongoing users with new users in the same analysis [15]. Thus, based on the current knowledge, it is unclear whether allopurinol treatment is independently associated with better cardiac outcomes.

Gout is commonly associated with several comorbidities, such as hypertension, diabetes etc. One-third of gout patients have diabetes [16]. Diabetes, as a major cardiovascular disease risk factor, affects 26 million Americans, is among the top 10 causes of death in the U.S., and cost $245 billion in direct and indirect costs in 2012 [17]. Despite the common occurence of both conditions and associated morbidity, studies of cardiovascular outcomes in patients with gout and diabetes are lacking.

We were interested in investigating cardiovascular (CV) events in patients with gout and concomitant diabetes, since this group of patients is at high risk of CV events, given that diabetes is an established CV risk factor and hyperuricemia (the key underlying abnormality in gout) has been implicated as a CV risk factor [1, 4–7]. Therefore our study objectives were to assess whether in patients with gout with diabetes, allopurinol therapy was associated with cardiovascular benefit as demonstrated by a lower incidence of acute cardiovascular events (myocardial infarction [MI] or stroke).

## Methods

Study methods and results are reported as recommended by the Strengthening of Reporting in Observational studies in Epidemiology (STROBE) statement [18].

### Study cohort and participants

The Institutional Review Board at the University of Alabama at Birmingham (UAB) approved the study. We conducted a retrospective cohort study using the Multi-Payer Claims Database (MPCD) that linked health plan data from national commercial and governmental insurances, representing beneficiaries with United Healthcare, Medicare, or Medicaid coverage during 2007–2010. MPCD data contain patients’ demographic and insurance coverage information from enrollment files, claims for inpatient and outpatient services, and prescription medications.

For this analysis, we examined patients who had a diagnosis of both gout and diabetes. Gout was defined as the occurrence of an inpatient admission or 2 outpatient visits with a physician encounter at least 7 days apart and the presence of International Classification of Diseases, ninth revision, clinical modification [ICD-9-CM] codes 274.xx for gout, shown to have a positive predictive value >90% [19]. Diabetes was ascertained by the presence of two ICD-9-CM codes of 250.xx associated with physician encounter visits at least 7 days apart but ≤365 days and the use of a diabetes-specific medication [20], also a validated definition with high positive predictive value >90% [21–23]. The person was diabetes-eligible and gout-eligible “completely” the day he/she satisfied both diseases’ eligibility criteria. The index date (follow up start) of the study cohort was defined as the day when the criteria for both disease eligibility and the 365 days of continuous coverage were satisfied. The baseline period was defined as 365 days before the start of follow up for each eligible participant episode. An individual was allowed to have more than 1 episode satisfying the 365 days of continuous coverage and at least 1 day for follow up, a standard methodology to maximize use of all data in the incident user design.

### Study outcome

The study outcome of interest was incident acute cardiovascular event, a composite of incident hospitalized MI or incident hospitalized stroke, in patients with gout and diabetes. We excluded patients with MI, stroke or coronary artery disease (CAD) in the baseline 365-day period for these analyses, using diagnoses for incident or prevalent cardiovascular disease (410, 412, 430–438, 428, 429.2x) (Additional file 1), in order to analyze the first incident MI and the first incident stroke.

Incident MI and incident stroke were defined as inpatient hospitalization with respective ICD-9-CM codes for MI or stroke with at least 1-day hospital stay, unless death occurred (Additional file 1). This approach is accurate for case identification with high positive predictive value >90% for these diagnoses and other cardiovascular risk factors [23–25].

We censored observation if there was an outpatient diagnosis of 410.x1 or an inpatient or outpatient diagnosis of 410 other than 410.x1 and 412 in the MI analyses but allowed for a 2-day “grace period” in which MI hospitalizations were sought. For example, someone with an outpatient diagnosis of MI who was hospitalized for it within 24 h with hospital length of stay >1-day would have met the MI definition. For stroke analyses, we censored patients with grace period of 2-days after the occurrence of an outpatient diagnosis of stroke (430.xx, 431.xx, 433.x1, 436.xx, 434.xx except 434.x0) or an inpatient or outpatient diagnosis of 430–438 other than that of stroke ICD-9-codes (listed above). This approach was used due to uncertainties about the clinical relevance of an outpatient diagnosis of MI or stroke and to exclude prevalent cases of MI or stroke.

### Exposure of interest

The study exposure of interest was the new use of allopurinol, defined based on a filled prescription for allopurinol, with no prior filled prescription of allopurinol for previous 183 days. We considered exposure days of medications from 2007 to 2010 allowing up to 30 days of stock to be carried over (if the medication was refilled slightly early). We allowed the days of supply of allopurinol to be a maximum of 120 days only. A new medication episode started if a patient had complete coverage but no filled prescription of allopurinol in previous 183 days. For the main analysis that used a new user design, we defined various time-varying allopurinol use categories for each person-day of follow-up as either current use (present day covered by allopurinol) or prior use (present day not covered by allopurinol, but prior period with allopurinol exposure) based on medication episodes. Each patient could contribute person-time to each of these groups, as per standard methodology in an incident user design, to allow maximal use of data.

### Covariates and confounders

Study covariates measured in the baseline period were age, gender and race/ethnicity. We categorized age in 5-year age group categories and race/ethnicity into Asian, Hispanic, Black, White, other and missing. Cardiovascular risk factors other than diabetes (hypertension, hyperlipidemia), and other medical comorbidities, including chronic obstructive pulmonary disease (COPD) and renal failure were assessed based on the presence of respective ICD-9-CM codes during the study period prior to occurrence of outcome, as time-varying variables (Additional file 1). Comorbidities were categorized as present or absent.

### Statistical analyses

We calculated summary statistics as proportion or mean (standard deviation). Unadjusted comparisons were performed using chi-square or t-tests, as appropriate. Crude incidence rates for MI and stroke were calculated for current and prior allopurinol users. We used the new user design as our main analysis to assess the association of allopurinol use with outcomes of interest [15].

We performed unadjusted and age-adjusted Cox regression analyses to obtain hazards of the composite acute cardiovascular event (incident MI or incident stroke) with allopurinol use. Multivariable-adjusted Cox regression analyses were performed next to account for demographics, cardiovascular risk factors (hypertension, hyperlipidemia), and comorbidities (COPD, renal failure). Hazard ratios for incident acute cardiovascular event were calculated, based on allopurinol use, current, vs. prior (referent). We performed statistical analyses using SAS version 9.2. A *p*-value <0.05 was considered significant.

We performed several sensitivity analyses to test the robustness of findings by: (1) additionally adjusting for autoimmune diseases, a potential risk factor for MI/stroke due to associated chronic inflammation; (2) additionally adjusting for the use of colchicine, a commonly used anti-inflammatory drug for gout that might have a cardio-protective effect [26]; or (3) using a prevalent user design, where we defined each day of follow-up as current, prior or never allopurinol use by adding never allopurinol users, defined as no allopurinol use (referent) from 2007 till the end of follow up.

## Results

### Patient demographic and clinical characteristics

We observed 2,053,185 person days of current allopurinol use and 1,671,583 person days of prior allopurinol use. Age distribution across groups was similar; 70.7% of current and 61.5% of prior allopurinol users were White (Table 1). Comorbidities were common in both groups: Hypertension, 85% each; hypercholesterolemia, 78–81%; and renal disease, 25–28% (Table 1).

**Table 1:** Characteristics of allopurinol users

	All combined (%)	Allopurinol use (%)	
		Current‡	Previous‡
Total person days	3,724,768	2,053,185	1,671,583
Male gender	53.76	54.00	53.47
Age group			
≤ 50	7.55	6.54	8.79
51–60	12.86	11.66	14.33
61–65	9.70	9.32	10.17
66–70	24.00	24.68	23.16
71–75	19.73	20.12	19.26
76–80	14.16	14.41	13.86
> 80	12.00	13.27	10.44
Race			
Asian	4.49	4.29	4.73
Black	19.68	16.83	23.18
Hispanic	2.61	2.01	3.35
Other	1.87	1.78	1.98
Missing	4.89	4.26	5.67
White	66.46	70.83	61.09
Comorbidity			
Hypertension	85.57	85.35	85.85
COPD	9.12	9.00	9.27
Renal disease	26.11	27.71	24.14
PVD	8.12	8.17	8.05
Hyperlipidemia*	79.56	80.53	78.38

‡ Characteristics of individuals that contributed at least 1 person day to that column, and people could be represented in both columns, if they contributed to both current and previous allopurinol use; *Hyperlipidemia was defined as statin use or an ICD-9-CM code for hypercholesterolemia

### Association of allopurinol use with incident MI or incident stroke

There were 158 and 151 incident acute cardiovascular events (MIs or strokes) in current and prior allopurinol users, respectively (Table 2). The crude rate of incident acute cardiovascular events (MI or stroke) in current and prior allopurinol users were 2.81 and 3.30 per 100 person-years, respectively. Association of allopurinol use with incident acute cardiovascular events (MI or stroke) in unadjusted and age-adjusted analyses including other significant correlates is shown in Table 3.

**Table 2:** New Allopurinol use and Incident MI^a^ or incident stroke^b^ outcome

Allopurinol use	Total Person days (total person years)	Incident MI or Stroke	Per 100,000 PD (per 100 PY)
Current	2,053,185​ person days (5621.3 person years)	158	7.70 (2.81 per 100 PY)
Previous	1,671,583​ person days (4576.5 person years)	151	9.03 (3.30 per 100 PY)

*PD* person-days; *PY* person years

^a^For MI, the person days with baseline 410, 412, 430–438, 428.xx and 429.2X were removed. Also, the person days were censored at the occurrence of first MI or an outpatient diagnosis of 410.X1 or inpatient or outpatient diagnosis of 410 except 410.x1 and 412

^b^For stroke, the person days with baseline 410, 412, 430–438, 428.xx and 429.2X were removed. Also, the person days were censored at the occurrence of first stroke or an outpatient diagnosis of stroke or inpatient or outpatient diagnosis of 430–438 except for the dx codes for stroke. Stroke (430.xx, 431.xx, 433.x1 (433.01, 433.11, 433.21, 433.31, 433.81, 433.91), 434.xx excluding 434.x0 (434.01, 434.11, 434.91), 436.xx

**Table 3:** Unadjusted and age-adjusted association of current allopurinol use with Incident acute cardiovascular event composite (MI or stroke) among patients with diabetes and gout

	Unadjusted		Age-adjusted	
	Hazard ratio (95% CI)	*p*-value	Hazard ratio (95% CI)	*p*-value
Allopurinol user				
Current	**0.71 (0.56, 0.89)**	**0.003**	**0.67 (0.53, 0.85)**	**0.0007**
Previous	Ref		Ref	
Male Gender	0.85 (0.68, 1.06)	0.14	1.01 (0.80, 1.27)	0.95
Age				
≤ 50	**0.40 (0.17, 0.92)**	**0.03**	-	-
51-60	0.86 (0.49, 1.50)	0.59	-	-
66-70	0.88 (0.54, 1.45)	0.62	-	-
71-75	**1.64 (1.03, 2.61)**	**0.04**	-	-
76-80	1.58 (0.97, 2.59)	0.07	-	-
> 80	**2.70 (1.70, 4.30)**	**<0.0001**	-	-
61-65	Ref		-	-
Race				
Asian	1.05 (0.62, 1.78)	0.85	0.95 (0.56, 1.60)	0.84
Black	0.90 (0.67, 1.21)	0.50	1.03 (0.77, 1.39)	0.84
Hispanic	1.35 (0.73, 2.47)	0.34	1.54 (0.84, 2.82)	0.16
Other	1.53 (0.79, 2.98)	0.21	1.59 (0.82, 3.10)	0.17
Missing	0.58 (0.30, 1.13)	0.11	0.73 (0.37, 1.43)	0.36
White	Ref		Ref	
Comorbidity				
Hypertension	1.08 (0.78, 1.50)	0.63	1.01 (0.73, 1.41)	0.93
Hyperlipidemia	0.90 (0.69, 1.17)	0.43	0.91 (0.69, 1.19)	0.49
COPD	1.10 (0.76, 1.59)	0.63	1.09 (0.75, 1.58)	0.66
Renal disease	**1.40 (1.11, 1.78)**	**0.005**	**1.34 (1.06, 1.70)**	**0.02**
Peripheral vascular disease	1.08 (0.73, 1.60)	0.70	0.96 (0.65, 1.43)	0.86
Immune diseases	1.05 (0.80, 1.39)	0.71	1.07 (0.81, 1.41)	0.62
Colchicine use	0.74 (0.51, 1.09)	0.13	0.77 (0.53, 1.12)	0.18

Significant odds ratios are in bold

*COPD* chronic obstructive pulmonary disease

In multivariable-adjusted models that accounted for demographics and cardiovascular risk factors, compared to prior users, current allopurinol users had significantly lower hazard of incident stroke or MI, HR was 0.67 (95% CI, 0.53, 0.84) (Table 4). Other significant risk factors were older age and renal disease (Table 4).

**Table 4:** Multivariable-adjusted associations of allopurinol use with incident composite outcome (MI and stroke)

	Incident MI or stroke	
	Hazard ratio (95% CI)	*p*-value
Allopurinol User		
Current	**0.67 (0.53, 0.84)**	**0.0006**
Previous	Ref	
Gender		
Male	1.00 (0.80, 1.26)	0.99
Female	Ref	
Age, in years		
≤ 50	**0.40 (0.17, 0.93)**	**0.03**
51–60	0.85 (0.49, 1.49)	0.57
66–70	0.88 (0.54, 1.45)	0.61
71–75	**1.64 (1.02, 2.62)**	**0.04**
76–80	1.59 (0.97, 2.60)	0.07
> 80	**2.70 (1.69, 4.34)**	**<0.0001**
61–65	Ref	
Race		
Asian	0.92 (0.54, 1.55)	0.74
Black	0.97 (0.72, 1.31)	0.84
Hispanic	1.41 (0.76, 2.59)	0.27
Other	1.55 (0.79, 3.03)	0.20
Missing	0.71 (0.36, 1.40)	0.32
White	Ref	
Comorbidity		
Hypertension	0.96 (0.69, 1.35)	0.83
Hyperlipidemia	0.91 (0.69, 1.20)	0.51
COPD	1.07 (0.74, 1.55)	0.73
Renal disease	**1.36 (1.07, 1.73)**	**0.01**
PVD	0.93 (0.63, 1.39)	0.74

Significant odds ratios are in bold

*PVD* peripheral vascular disease, *COPD* chronic obstructive pulmonary disease

### Sensitivity analyses: adjustment for colchicine or immune disease and by prevalent use

Sensitivity analyses adjusting for colchicine revealed essentially the same results for current allopurinol use (yes/no) as in the main analyses above (Table 5). Colchicine was not significantly associated with the risk of incident MI or stroke, 0.80 (95% CI, 0.55, 1.18) (Table 5). Sensitivity analyses, additionally adjusted for immune disease confirmed the findings from the main analyses (Table 5); the presence of immune disease was not significantly associated, 1.04 (95% CI, 0.78, 1.37).

**Table 5:** Sensitivity analyses with inclusion of colchicine in the main model or with immune disease in the model

	Main model^a^ + colchicine		Main model^a^ + immune disease	
	Incident MI or stroke			
	Hazard ratio (95% CI)	*p*-value	Hazard ratio (95% CI)	*p*-value
Allopurinol User				
Current	**0.68 (0.54, 0.85)**	**0.001**	**0.67 (0.53, 0.84)**	**0.0006**
Previous	Ref		Ref	
Immune disease	--	--	1.04 (0.78, 1.37)	0.81
Colchicine	0.80 (0.55, 1.18)	0.26	--	**--**

Significant odds ratios are in bold

^a^Main model was run without immune disease and without colchicine and included gender, age, race, hypertension, hyperlipidemia, peripheral vascular disease COPD and renal disease

In multivariable adjusted analyses that used a prevalent user design analysis (not as robust as incident user design), we found that current allopurinol use was associated with similar hazard reduction of incident MI or stroke (as in the main analyses) when compared to: (1) prior allopurinol use, 0.84 (95% CI, 0.72, 0.98); and (2) never use, 0.86 (95% CI, 0.75, 0.99) (Additional file 2).

## Discussion

In this study, we found that current use of allopurinol was associated with a statistically significant and clinically meaningful reduction of the risk of acute cardiovascular events (incident stroke or MI) in patients with gout and diabetes. We also provided crude incident rates for acute cardiovascular events in patients with gout and diabetes, by current versus prior allopurinol use. Several findings in this study deserve further discussion.

Our finding of 33% hazard reduction of incident stroke or MI with current allopurinol use compared to prior allopurinol use in patients with gout and diabetes is novel. We are unaware of other large studies that have examined this question. We chose to study this patient population (gout and diabetes) due to a higher risk of cardiovascular events. The beneficial cardiovascular effect of allopurinol may be related to several potential mechanisms including: (1) allopurinol associated attenuation of intercellular adhesion molecule-1 levels noted in patients after a recent stroke [27]; (2) improvement in endothelial function and reduction of oxidative stress with allopurinol [28–33]; and (3) reduction in glycosylated hemoglobin (HbA1C) with high-doses of allopurinol [33]. The potential mechanisms of cardiovascular risk reduction with reduction of serum urate, including oxidative stress and inflammation, were recently reviewed [34]. The hazards were decreased by 29% in the prevalent user analyses. This is consistent with a greater risk reduction seen in the incident user vs. prevalent user design, and might explain the lack of finding an association if prevalent user design is used and the sample size is smaller or events fewer.

Our findings must be interpreted in the context of current knowledge in this area. Most previous studies examined stroke as part of a composite cardiovascular outcome that included multiple cardiovascular outcomes, not just stroke and MI. In a 7-year follow-up of a 2-year RCT in patients with chronic kidney disease with glomerular filtration rate of < 60 ml/min, adjusted for race, sex and renal function, allopurinol use was associated with lower risk of 0.43 (95% CI, 0.21–0.88) of cardiovascular events (including CAD, cerebrovascular disease, heart failure and peripheral vascular disease) [35]. In an observational study of 187 patients with hypertensive nephropathy, allopurinol use was associated with a reduced hazard of 0.34 (*p* = 0.04) for cardiovascular disease (CAD, heart failure and stroke) [36]. In a study using Scottish database, allopurinol use was associated with no difference in adjusted hazards of cardiovascular events (non-fatal myocardial infarction, non-fatal stroke and cardiovascular mortality) compared to non-users 1.10 (95% CI, 0.95–1.26) [14]. Differences in the conditions included in the composite cardiovascular outcome, study populations and study designs, likely explain the differences in findings.

The three key studies examining the risk of MI reported that compared to non-use, allopurinol use was associated with a 48% decrease [13] vs. a 25% increase [14] vs. no significant change [12] in the risk of MI. The studies were similar in that they all used clinical databases, assessed non-fatal MI and controlled for several cardiovascular risk factors. However, several differences were evident. Two studies used prevalent-user design [12, 14], which puts them at the risk of adjusting for the mediating factors and missing early events. Thus, it was not surprising that the two studies that used prevalent design showed increased risk [14] or no difference in risk [12], while the only new user design study showed risk reduction for MI [13]. Our study, using a new user design, also shows statistically significant benefits of allopurinol for risk reduction of acute cardiovascular events, and these findings were confirmed in several sensitivity analyses, including an analysis of prevalent allopurinol users (to allow comparability to the two previous studies). Our study is among the first to show allopurinol’s protective effect on acute cardiovascular event, i.e., incident stroke or MI, in patients with gout and diabetes. However, our study differs in one important aspect from the previous cited studies, i.e., we focused on patients with both gout and diabetes. These patients likely have a higher risk of acute cardiovascular events than patients with only one of the two risk factors. This likely enriched our population for more acute cardiovascular events and likely made our study adequately powered, as intended.

This finding has a practical implication for patients and providers. For patients with both gout and diabetes, allopurinol has an added benefit of reduction of the risk of acute MI or stroke. The risk reduction is not only statistically significant, but also clinically meaningful (33% reduction). Patients should be informed of this potentially beneficial effect, when allopurinol is first prescribed. This risk reduction may potentially be even greater in patients with other cardiovascular disease risk factors.

Our study findings must be interpreted considering several limitations. The diagnosis of acute cardiovascular events, our study outcome, was based on the presence of ICD-9-CM codes, which may lead to misclassification bias. However, previous studies have shown the these codes for cardiovascular outcomes were accurate [24]. There is also evidence of a reasonable accuracy for codes used for covariates i.e., cardiovascular risk factors [23–25]. Serum urate levels were available for a small proportion of patients and therefore, we could not examine whether these associations were or were not mediated via the reduction of serum urate levels. The database also did not allow us to include other variables such as body mass index, smoking, diet and exercise, over the counter aspirin use, and family history of cardiovascular disease, important covariates/risk factors for MI and stroke. We had considered excluding patients with cancer, but decided to include them in our study to improve the generalizability of study findings. A very small proportion of patients on allopurinol (<0.5%) are usually receiving short-term allopurinol for prophylaxis against tumor lysis syndrome, since they are receiving chemotherapy [37]; we doubt that this study limitation influenced our results significantly. Examination of effect of allopurinol on other outcomes, including renal failure, dialysis or death, or rare side effects such as allergic reactions, was beyond the scope of current study. Future studies should examine these outcomes.

The contribution of healthy adherer effect to the observed allopurinol’s beneficial effect for these outcomes can not be ruled out, since patients adherent to/using allopurinol may have other healthy behaviors, such as high adherence to other medications, healthier lifestyle, preventive care etc., which may all reduce the risk of stroke and MI. Whether these findings can be generalized to populations other than diabetes and gout is not known, and further study is needed in this area. Reasons for allopurinol discontinuation can be other than non-adherence, such as allopurinol cessation due to severe renal disease (which is not a contraindication for allopurinol use, since allopurinol can be used even in patients on severe kidney dysfunction and also dialysis), suspected or confirmed allergic reactions (which are extremely rare), the concomitant/new use of azathioprine for the rheumatic disease or organ transplantation (which is uncommon/rare), or switching to febuxostat, another xanthine oxidase inhibitor launched in 2009 (which would have likely decreased the observed difference, but likely did not have a major impact given the use of febuxostat in <7% patients with gout) [38]. These confounding biases would have likely reduced the differences, and likely made our current estimates conservative, since most of these conditions would likely increase the risk of MI and/or stroke in current allopurinol non-users.

Study strengths include a large sample size, the use of a sample that allows findings to be generalizable to cohorts with gout or diabetes in the U.S., the use of a new user design that reduces bias in observational studies [39–41], the inclusion of multiple risk factors for MI/stroke, and the robustness of estimates in sensitivity analyses.

## Conclusions

In conclusion, in robust analyses of data from a representative sample of Americans, we found that current allopurinol use was independently associated with a lower risk of incident stroke or MI in patients with gout and diabetes. Our findings provide evidence for a potential cardiovascular protective effect of allopurinol in patients with gout and diabetes, a group of patients at high risk for cardiovascular events. Future studies need to explore the factors that mediate this protective effect, and examine what proportion of variability in this protective effect is explained by serum urate reduction and/or anti-oxidant effect of allopurinol.

## Additional files

Additional file 1: International Classification for Diseases, Ninth revision, Clinical Modification (ICD-9-CM) Diagnostic Codes for each condition used for outcome, cohort eligibility, and covariate definitions. This file provides the ICD-9-CM codes for underlying conditions, study outcomes and covariates. (DOC 43 kb)
Additional file 2: Multivariable-adjusted associations of allopurinol use with composite outcome (MI or stroke) for prevalent allopurinol users* with gout and diabetes, with previous allopurinol users as the reference category. This file provides the results of sensitivity analyses, repeating the main analysis in prevalent allopurinol users, instead of incident allopurinol users. (DOC 31 kb)

## Abbreviations

COPD: Chronic obstructive pulmonary disease
CV: Cardiovascular
ICD-9-CM: International Classification of Diseases, ninth revision, clinical modification
MI: Myocardial infarction
MI: Myocardial infarction
MPCD: Multi-Payer Claims Database
STROBE: Strengthening of Reporting in Observational studies in Epidemiology
UAB: University of Alabama at Birmingham
