# Supplementary material for: Allopurinol use and the risk of acute cardiovascular events in patients with gout and diabetes
Source: BMC Cardiovasc Disord. 2017 Mar 14;17:76. doi: 10.1186/s12872-017-0513-6 (PMC5348884; doi:10.1186/s12872-017-0513-6)
Supplement: Additional file 1: — International Classification for Diseases, Ninth revision, Clinical Modification (ICD-9-CM) Diagnostic Codes for each condition used for outcome, cohort eligibility, and covariate definitions. This file provides the ICD-9-CM codes for underlying conditions, study outcomes and covariates. (DOC 43 kb) [file 12872_2017_513_MOESM1_ESM.doc]

**Additional file 1.** International Classification for Diseases, Ninth revision, Clinical Modification **(**ICD-9-CM) Diagnostic Codes for each condition used for outcome, cohort eligibility, and covariate definitions

|  | **ICD-9-CM Codes** |
| --- | --- |
| **Study Outcome: Incident myocardial infarction (MI) or stroke** |  |
| Incident MI | 410.x1: 410.01, 410.11, 410.21, 410.31, 410.41, 410.51, 410.61, 410.71, 410.81, 410.91 |
| Incident stroke | 430.xx: all codes with 430 in the first 3 places  431.xx: all codes with 431 in the first 3 places  433.x1: 433.01, 433.11, 433.21, 433.31, 433.41,433.51, 433.61, 433.71, 433.81, 433.91  434.xx except 434.x0: all codes with 434 in the first 3 places except the ones with 0 in the last place  436.xx: all codes with 436 in the first 3 places |
|  |  |
| **Disease cohort: Gout and Diabetes** |  |
| Gout | 274.xx |
| Diabetes | 250.xx |
|  |  |
| **Baseline covariates** |  |
| Hypertension | 401.xx - 405.xx |
| Chronic obstructive pulmonary disease (COPD) | 491.xx, 492.xx, 496.xx |
| Chronic kidney disease | 582.xx, 583.xx, 585.xx, 586.xx, 588.xx |
| Peripheral vascular disease (PVD) | 440.20-440.24, 440.31-440.32, 440.8, 440.9, 443.9, 785.4,V434, 441.xx |
| Statin use | No code; from medication fill data |
| Hyperlipidemia | 272.0, 272.1, 272.2, 272.3, 272.4 |
| Autoimmune disease | 720.xx (Ankylosing spondylitis and other inflammatory spondylopathies), 283.xx (hemolytic anemia), 579.xx (Intestinal malabsorption), 255.(disorders of adrenal gland) , 242.xx (thyrotoxicosis with or without goiter), 357.xx (inflammatory and toxic neuropathy), 555.xx (regional enteritis), 556.xx (ulcerative colitis), 359.xx (muscle dystrophies and other myopathies), 340.xx (multiple sclerosis), 358.xx (myoneural disorders), 281.xx (Other deficiency anemias), 725.xx (Polymyalgia rheumatica), 696.xx (Psoriasis and similar disorders), 710.xx (Diffuse diseases of connective tissue), 255.4x (Cortcioadrenal insufficiency), 279.8x (Other specified disorders involving the immune mechanism), 694.5x (Pemphigoid**)**, 710.3x (Dermatomyositis), 245.2x (Chronic lymphocytic thyroiditis), 695.4x 9 (Lupus erythematosus), 694.4x (Pemphigus), 694.5x (Pemphigoid), 694.6x (Benign mucous membrane pemphigoid), 710.4x (Polymyositis), 571.6 9 (Biliary cirrhosis), 696.1 (Other psoriasis), 410.2x, 446.5x 9 (Giant cell arteritis), 446.4x (Wegener's granulomatosis), 323.61 (Infectious acute disseminated encephalomyelitis), 571.42 9 (Autoimmune hepatitis), 250.01 (Diabetes mellitus), 250.03 (Diabetes mellitus), 446.21 (Goodpasture's syndrome), 287.31 (Immune thrombocytopenic purpura), 258.01 (Multiple endocrine neoplasia type I), 362.18 (Retinal vasculitis) |
|  |  |
| **Baseline exclusion** | MI [410.xx or 412.xx], stroke [430-438] or heart disease [410-414, 428.xx and 429.2x] |
|  |  |
| **Censoring (includes prevalent cases and outpatient codes)** |  |
| Myocardial infarction (MI) | An outpatient code of 410.x1 or an inpatient or outpatient code of 410 other than 410.x1 and 412 |
| Stroke | Outpatient code for stroke (430.xx, 431.xx, 433.x1, 436.xx, 434.xx except 434.x0) or an inpatient or outpatient code of 430-438 other than that of stroke ICD-9- codes listed at the beginning of the sentence. |
