# Supplementary material for: Allopurinol use and the risk of acute cardiovascular events in patients with gout and diabetes
Source: BMC Cardiovasc Disord. 2017 Mar 14;17:76. doi: 10.1186/s12872-017-0513-6 (PMC5348884; doi:10.1186/s12872-017-0513-6)
Supplement: Additional file 2: — Multivariable-adjusted associations of allopurinol use with composite outcome (MI or stroke) for prevalent allopurinol users* with gout and diabetes, with previous allopurinol users as the reference category. This file provides the results of sensitivity analyses, repeating the main analysis in prevalent allopurinol users, instead of incident allopurinol users. (DOC 31 kb) [file 12872_2017_513_MOESM2_ESM.doc]

**Additional file 2.** Multivariable-adjusted associations of allopurinol use with composite outcome (MI or stroke) for prevalent allopurinol users* with gout and diabetes, with previous allopurinol users as the reference category

|  | **Incident MI or stroke** | | | | |
| --- | --- | --- | --- | --- | --- |
|  | **Hazard ratio**  **(95% CI)** | **p-value** |  | **Hazard ratio**  **(95% CI)** | **p-value** |
| **Allopurinol User** |  |  | **Allopurinol User** |  |  |
| **Current** | **0.84 (0.72, 0.98)** | **0.03** | **Current** | **0.86 (0.75, 0.99)** | **0.03** |
| **Never** | 0.98 (0.84, 1.15) | 0.82 | **Previous** | 1.02 (0.87, 1.19) | 0.82 |
| **Previous** | Ref |  | **Never** | Ref |  |

*Prevalent allopurinol users are defined at the study beginning, without any baseline period

Two hazard ratios in the table are with two different reference categories, one with previous use of allopurinol and one with never use.
